# Study of the influence of nanoscale porosity on the microbial electroactivity between expanded graphite electrodes and *Geobacter sulfurreducens* biofilms

**DOI:** 10.1111/1751-7915.14357

**Published:** 2023-12-27

**Authors:** M. Ramírez‐Moreno, R. Berenguer, J. M. Ortiz, A. Esteve‐Núñez

**Affiliations:** ^1^ Bioe Group, Instituto Madrileño de Estudios Avanzados IMDEA‐Agua Parque Tecnológico de la Universidad de Alcalá Alcalá de Henares Spain; ^2^ Departamento de Química Analítica, Química Física e Ingeniería Química Universidad de Alcalá Alcalá de Henares Spain; ^3^ Departamento de Química Física, Instituto Universitario de Materiales Universidad de Alicante Alicante Spain

## Abstract

Expanded graphite (EG) electrodes gather several advantages for their utilization in microbial electrochemical technologies (MET). Unfortunately, the low microbial electroactivity makes them non‐practical for implementing them as electrodes. The objective of this work is to explore the enhancement of microbial electroactivity of expanded graphite (commercial PV15) through the generation of nanopores by CO_2_ treatment. The changes in properties were thoroughly analysed by TG, XRD, Raman, XPS, gas adsorption, SEM and AFM, as well as microbial electroactivity in the presence of *Geobacter sulfurreducens*. Nanopores remarkably enhance the microbially derived electrical current (60‐fold increase). Given the inaccessibility of micron‐sized bacteria to these nanopores, it is suggested that the electric charge exchanged by electroactive microorganisms might be greatly affected by the capability of the electrode to compensate these charges through ion adsorption. The increased microbial current density produced on activated PV15 opens the possibility of using such materials as promising electrodes in MET.

## Graphical abstract

Electric current density generated by *Geobacter sulfurreducens* and proposed effect of nano‐scale porosity in the microbial extracellular electron transfer.

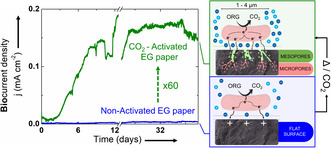

## INTRODUCTION

Global challenges in the water‐energy‐climate nexus demand the development of new technologies and energy sources (UNESCO/UN‐Water, 2020). In this context, the emergence of microbial electrochemical technologies (METs) is receiving growing interest. These technologies use electroactive microorganisms that can exchange electrons with a conductive and/or electroactive material (Lovley, 2006), in most cases, to convert the chemical energy contained in organic compounds into electric energy and/or valuable inorganic and organic chemicals (Logan & Rabaey, 2012), or just to promote microbial metabolism. The practical utilization of these microorganisms is an emerging field that is giving rise to different applications, including energy production (Logan, 2009), wastewater treatment (Aguirre‐Sierra et al., 2020), electrobioremediation (Wang et al., 2020), bioelectrosynthesis (ter Heijne et al., 2019), biosensors (Chung et al., 2020), desalination (Cao et al., 2009; Ramírez‐Moreno et al., 2019) among others.

Over the last two decades, the research in this field has proved that the nature of the electrode materials plays a key role in determining microbial electroactivity (Haluk Beyenal, 2005; Maestro et al., 2014; Prado et al., 2019) and, therefore, the overall system performance for these applications. In addition, the electrode material is one of the critical factors in determining the cost and sustainability of MET (Rozendal et al., 2008).

Among several candidates, the state‐of‐the‐art for MET is generally based on highly conductive carbon electrodes. These materials typically exhibit good stability, biocompatibility and a well‐developed graphitic structure that ensures a high electrical conductivity, being this property essential for generating electrical power in different types of MET. Examples of these most used conductive carbon electrodes for electromicrobial applications are generally 3D conformations, dense (sheets, rods, plates, etc.) or porous (papers, felts, cloths, foams, etc.), of graphite, carbon fibres and glassy carbon (Alvarez Esquivel et al., 2020; Logan, 2010). Generally, the dense conformations are prone to provide higher conductivities, while the porous ones expose a higher accessible surface area for extended biofilm growth, which results in larger microbial currents (Chong et al., 2019). These 3D porous materials, however, usually suffer from clogging, internal acidification and/or unstable responses during operation, among other drawbacks. Hence, for microbial electrochemical applications, it would be desirable to attain the high conductivities of the dense electrode conformations together with the enhanced microbial activities of the porous ones.

In this context, the study of novel approaches and/or strategies to optimize the electrode response in METs becomes key for the implementation of microbial electrochemical technology in real applications. Thus, several modification treatments have been attempted to improve the microbial electroactivity of carbon electrodes, and most of them have been devoted to increasing the conductivity and external surface area. Nonetheless, apart from these properties, carbon materials can exhibit characteristic‐rich surface chemistry and nano‐sized porous structure, including micropores (*d*, pore diameter; *d* < 2 nm) and mesopores (2 < *d* < 50 nm), which play a critical role in other electrochemical technologies without bacteria, such as energy storage and conversion, environmental remediation, etc. (Liu & Creager, 2010; Momodu et al., 2017; Zhang et al., 2014a). These extraordinary effects of atomic species and nanoscale features can be explained by the fact that they can directly interact with electrons and ions, the main actors in electrochemical processes.

In the case of MET, the influence of electrode porosity on microbial performance has been scarcely studied until now. Thus, Chen et al. reported that microporous‐ and mesoporous‐activated carbon, used as a bioanode in a microbial fuel cell (MFC), improves the performance since this nanoscale structure could promote charge transfer and microbial adhesion (Chen et al., 2018). In this sense, it is generally thought that the micro‐ and meso‐porosity, which cannot host micron‐sized bacteria, cause any direct effects on the performance of MET (Chong et al., 2019), as the surface is not accessible by microorganisms. However, recent works on the so‐called METlands®, for which current production is not essential, have evidenced the better microbial electrochemical performance of some biochars, materials with comparatively much poorer conductivity (Prado, Ramírez‐Vargas, et al., 2020). These findings have led researchers to hypothesize that the large volume of micropores in these materials could enhance the activity of electroactive microorganisms (Berenguer et al., 2020; Schievano et al., 2019). Hence, the study on the influence of nano‐scaled porosity of electrodes in the performance of MET is still an unexplored topic with a huge potential impact on this emerging field.

To face this study, ideally, it is necessary to compare carbon materials in which the only difference must be the nanoporosity to avoid any potential interference of other intercorrelated properties, such as surface chemistry, microstructure or conductivity. In fact, it is well known that the change of nanoporosity usually alters these properties. For the aforementioned reason, a suitable choice of carbon material and the modification technique are necessary to precisely and systematically change the nanoporosity. Furthermore, the choice of carbon material with real applicability in MET may greatly contribute to highlighting the potential of this study.

Particularly, expanded graphite (EG) is a carbon material commonly used in various electrochemical applications/devices, mainly as a bipolar electrode or current collector (Guo et al., 2021; Kim et al., 2021). This is a relatively low‐cost material exhibiting great corrosion resistance, high electrical conductivity and density, as well as a matchless simplicity of handling and adaptation to most electrochemical cells. All these features of EG are important advantages for its utilization in MET. However, it is practically a smooth material with negligible specific surface area, so its predictable low microbial electroactivity may have made it practically useful only as a current collector (e.g. in combination with graphite felt). This may also explain why there are few studies analysing the performance of EGs in microbial electrochemical systems (Alvarez Esquivel et al., 2020; Rajendran et al., 2022).

This work explores the impact of nanoscale porosity from a commercial EG electrode on the electroactivity of *Geobacter sulfurreducens* (1–4 μm size), a model electroactive microorganism (Bond & Lovley, 2003; Ishii et al., 2008; Marsili et al., 2008; Speers & Reguera, 2012). For this purpose, first, the physical activation of EG with CO_2_ was investigated, and the effects of activation temperature and time were analysed. Next, the physicochemical and electrochemical properties of the EGs, before and after CO_2_ activation, were characterized by several techniques. Finally, the microbial electroactivity of selected materials was evaluated through cyclic voltammetry and chronoamperometry techniques in a three‐electrode bioreactor with a pure *Geobacter sulfurreducens* culture and acetate as an electron donor. Moreover, scanning electron microscopy (SEM) and fluorescence laser scanning microscopy (LSM) were used to visualize the colonization and metabolic activity of biofilms on the studied electrodes.

## EXPERIMENTAL PROCEDURES

### Materials

A commercial EG from SGL‐Carbon, called PV15 (SIGRACELL® bipolar plates), was chosen for this study. PV15 materials are flexible, thin (0.6 mm) and flat sheets of fluoropolymer‐bonded expanded graphite with a low weight footprint. The electrical resistivity of this material is around 7 × 10^−4^ Ω cm (in parallel to the surface). Another type of EG was used in this work for comparison purposes: flexible graphite *Papyex®* (from Mersen), with electrical resistivity of 1 × 10^−3^ Ω cm (parallel). Finally, an isostatically pressed graphite plate (from Mersen) was used as a control electrode in the growth electroactive biofilm study. The electrical resistivity of this control material is 8 × 10^−4^ Ω cm (electrode thickness = 5 mm). Table 1 shows the characteristics of electrode materials provided by manufacturers.

**TABLE 1:** Characteristics of electrode materials provided by manufacturers.

Electrode	Company	Thickness	Electrical resistivity	Bulk density	Thermal conductivity
		(mm)	(Ω cm)	(g cm^−3^)	(W m^−1^ K^−1^)
PV15^a^	SGL Carbon	0.6	7 × 10^−4^ (in parallel) 3 × 10^−1^ (in perpendicular)	1.75	300
Papyex^b^	Mersen	1	1 × 10^−3^ (in parallel) 5 × 10^−2^ (in perpendicular)	0.7–1.3	Variable (50–150)
Graphite plate (grade 6503)^c^	Mersen	5	8 × 10^−4^	17.74	200

*Note*: Further details available in:

^a^ https://www.sglcarbon.com/en/markets‐solutions/applications/redox‐flow‐batteries/#.

^b^ https://www.mersen.co.uk/sites/uk/files/publications‐media/6‐gs‐papyex‐flexible‐graphite‐mersen.pdf.

^c^ https://www.mersen.co.uk/sites/uk/files/publications‐media/1‐markets‐energy‐solar‐carbon‐graphite‐photovoltaic‐mersen.pdf.

### Physical activation with CO_2_

PV15 foils with different porosities were prepared by physical activation (i.e. partial gasification) with CO_2_ at different temperatures and for distinct times. To do so, the foils were cut into pieces of 1.2 × 0.7 mm and introduced in the sample holder (alumina) of a simultaneous TGA/DSC 2 thermogravimetric system (Mettler‐Toledo), which enabled monitoring the sample weight‐loss during activation. The reactor was initially evacuated with N_2_ at room temperature for 10 min and then heated at 20°C min^−1^ under a continuous flow of 100 mL (STP) min^−1^ of N_2_: CO_2_ = 1:9 gas up to the desired activation temperature, ranging from 600 to 900°C. Next, the gasification experiments were carried out isothermally at these temperatures by using holding times ranging from 4 to 12 h. In this sense, longer treatments were not studied to avoid the formation of macropores (by excessive widening of porosity), thus, enabling this work to focus only on the effect of the smallest pores (Rodríguez‐Reinoso et al., 1995). The influence of both the temperature and time on the activation degree was studied. The obtained samples are referred to as *PV15‐T–t*, where *T* is the temperature (in °C), and *t* is the holding time (in h) in the isothermal treatment.

### Physicochemical characterization

The thermal behaviour of PV15 was analysed by thermogravimetry under both N_2_ and N_2_: CO_2_ = 1:9 gas using the same equipment to that of Section Physical activation with CO_2_. The textural properties of the different samples were characterized by gas adsorption together with the assistance of SEM and atomic force microscopy (AFM). N_2_ adsorption–desorption at 196°C and CO_2_ adsorption at 0°C were performed on a Quadrasorb‐Kr/MP apparatus (Quantachrome Instruments), after outgassing at 250°C under vacuum for 8 h. The specific surface area (S_BET_) and the total volume of micropores (V_DR_(N_2_)) (pore diameter (*d*) < 2 nm) were calculated according to the BET and the Dubinin–Radushkevich (DR) equations, respectively, from N_2_ adsorption/desorption isotherms (between 0.005 < *P*/*P*_0_ < 0.15) (Lozano‐Castello et al., 2009). The mesopore volume (*V*_mes_) (2 < *d* < 50 nm) was determined as the difference between the total pore volume (*V*_0.995_, volume at relative pressure of 0.995) and the micropore volume (V_DR_(N_2_)) (Lozano‐Castello et al., 2009). On the other hand, the volume of narrowest micropores (V_DR_(CO_2_)) (the so‐called ultramicropores, *d* < 0.7 nm) was derived from the adsorption of CO_2_ at 0°C also by using the DR equation (*P*/*P*_0_ < 0.025) (Lozano‐Castello et al., 2009). SEM images were obtained by using a JEOL JSM‐840 microscope operating at 15 kV, while topographic information was derived from AFM by using NTEGRA Prima equipment (NT‐MDT SPM).

X‐ray diffraction (XRD) measurements were obtained with the aid of a KRISTALLOFLEX K 760‐80F diffractometer (Bruker D8‐Advance) with a Ni‐filtered CuKα radiation (*λ* = 1.5416 Å) generated at 40 kV and 40 mA. The profile intensities were recorded stepwise within 2θ = 10–60° at a scan rate of 1° min^−1^ and with a scan step of 0.05° in 2θ (step time 3 s). Raman spectra were recorded with a Jasco NRS‐5100 dispersive system using a frequency‐doubled Nd:YAG laser at 532 nm, with a maximal spectral resolution of 1 cm^−1^, and a Peltier cooled CCD detector. The electrical conductivity measurements were carried out by using Lucas Lab resistivity equipment with four probes in‐line. In addition, the surface chemistry of the graphite foils was studied by X‐ray photoelectron spectroscopy (XPS) in a K‐Alpha spectrometer (Thermo‐Scientific) with MgKα radiation (1253.6 eV).

### Assembly, operation and electrochemical analysis of the bioreactor

**FIGURE 1:**
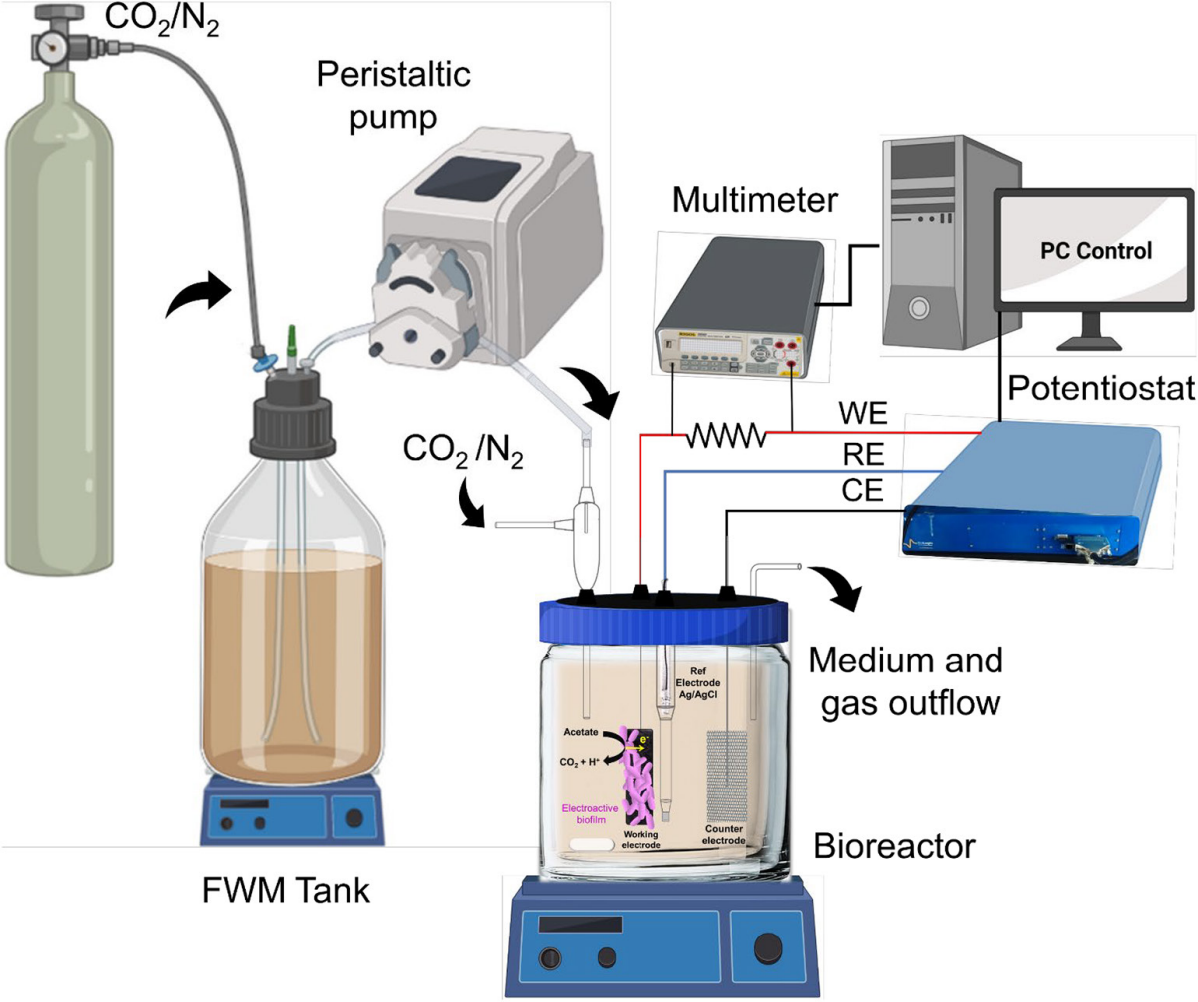
Schematic diagram of the laboratory assembly to study the growth and electroactivity of biofilms on the different studied materials used as working electrodes. Created with Biorender.com.

The microbial electroactivity of the different PV15 samples (activated and non‐activated) was studied in a single‐chamber bioreactor using a three‐electrode configuration. This experimental setup enabled simultaneous electrochemical control and measurement of the microbial response on different electrode materials under the same physicochemical and biological conditions, thus, ensuring a meaningful performance comparison (Prado, Berenguer, et al., 2020). The schematic diagram of the laboratory assembly is shown in Figure 1 and a photo of the real laboratory system setup is shown in Figure S1A.

The three‐electrode bioreactor was assembled into a sterilization hood to avoid contamination (Figure S1B). The sterilized bioreactor was filled with a 600 mL freshwater medium [FWM, pH = 7.4, electric conductivity (EC) = 6.2 mS cm^−1^] composed of vitamins, minerals (Esteve‐Nunez et al., 2005) and 20 mM acetate as an electron donor; the polarized electrode was the only electron acceptor. Three types of EG electrodes (PV15, Papyex and CO_2_‐activated PV15), as well as the isostatic graphite plate, were used as working electrodes. This last material is a well‐known carbonaceous surface used as a control electrode to confirm the appropriate behaviour of electroactive bacteria in the bioreactor. The counter electrode was a platinized titanium mesh, while the reference electrode was Ag/AgCl (3 M NaCl) (RE‐5B BASi, USA). A fritted glass chamber with 3 M NaCl was used as lugging capillary to place the reference electrode (Figure S1C,D). The potential of this reference electrode was checked prior to the experiments.

An optimal connection of the different electrodes is paramount to measuring meaningful and reproducible signals. According to previous works, this was ensured depending on its nature (Prado, Berenguer, et al., 2020). Details of these connections (Figure S1E,F) as well as the geometric surface area and resistance of anode electrodes with the connections are provided in Table S1.

After its assembly, the bioreactor was hydraulically connected with a sterilized feeding tank (2 L of FWM without electron acceptor) and electrically connected to a polarization and data acquisition instrument (BioLogic (SP‐150) potentiostat and Keithley Integra Series 2700 Multimeter, respectively) (Prado, Berenguer, et al., 2020) (Figure 1). Then, the whole system was purged with a gas phase of N_2_/CO_2_ (80%/20%) passing through an oxygen filter (Gas Clean Filter System, Agilent Technologies). Before inoculation of the bioreactor, initial CV (scan rates: 5 and 10 mV s^−1^) was performed (in FWM) to characterize the surface of each working electrode, ensure the proper connections and verify the current intensity absence from other analytes inside the bioreactor. After these abiotic CVs, all the electrodes were simultaneously polarized at +0.2 V (vs. reference electrode).

Then, the bioreactor inoculation was carried out by adding 25% (v/v) of a pure anaerobic exponential‐phase culture of *Geobacter sulfurreducens* (strain DL1), as previously reported (Ramírez‐Moreno et al., 2023). The culture was grown at 30°C in septum‐sealed serum bottles (50 mL working volume) in freshwater medium (FWM, pH = 6.9, electric conductivity (EC) at 25°C = 11.4 mS cm^−1^) containing the following salts: 2.5 g L^−1^ NaHCO_3_, 0.5 g L^−1^ NH_4_Cl, 0.6 g L^−1^ NaH_2_PO_4_.6H_2_O and 0.1 g L^−1^ KCl. Additionally, trace mineral and vitamin solutions were added (rate 1:100) (Esteve‐Nunez et al., 2005). Sodium acetate (C_2_H_3_NaO_2_, 20 mM) was used as a carbon source and the only electron donor, and disodium fumarate (C_4_H_2_Na_2_O_4_, 40 mM) was used as the sole electron acceptor. The culture media was degassed with a mixture of N_2_/CO_2_ (80:20, ALIGAL‐12, Air Liquide) bubbled in the serum bottle before inoculation. Possible traces of oxygen were removed from the gas phase by passing the gas through an oxygen filter (Gas Clean Filter System, Agilent Technologies). Exponential‐phase culture (with an optical density, at 600 nm, of 0.4) was used as inoculum in the bioreactor.

After inoculation, the bioreactor was initially operated in batch mode for the first 48 h, and then a constant flux of FWM (0.7 mL min^−1^) was circulated with a peristaltic pump from the sterilized feeding tank to the bioreactor. With this continuous operation mode, the FWM was renovated inside the bioreactor to maintain the electron‐donor substrate and avoid changes in pH due to the metabolism of electroactive bacteria. The current density evolution provided by each anode was recorded over time and calculated with the geometric area of the electrodes. The evolution of biofilm growth was tested by biotic CV at different times during the experiment. The potential window was between 0.8 V and −0.8 V (vs. Ag/AgCl; NaCl 3 M reference electrode), and the scan rate was 5 mV s^−1^. The bioreactor was continuously purged with N_2_/CO_2_ in the headspace, and the media were continuously stirred at a low rate. The temperature was maintained at 30–35°C in the bioreactor during the entire experiment. During the CV, the low agitation and pumping of the new medium into the reactor were not stopped. In addition, an abiotic control experiment (chronoamperometry without electroactive inoculum) of these activated electrodes was carried out (see Figure S5).

### Biofilms microscopy analysis

SEM (Digital Scanning Microscope DSM‐950) was used to visualize the surface morphology of electrodes. The electrode samples were submerged into a fixation solution (Cacodylate buffer, 0.2 M, pH 7.2, containing 5% glutaraldehyde) for 1 h at room temperature. The samples were rinsed in 0.2 M cacodylate buffer for 10 min and then dehydrated at room temperature in an ascending graded ethanol series (25%, 50%, 70%, 90% and 100%; 10 min each stage). Finally, the samples were rinsed in acetone for 10 min and immersed in anhydrous acetone at 4°C overnight. The last steps were carried out in the microscopy service of Alcalá University, where the dehydrated samples were dried in CO_2_ at the critical point. Also, they were mounted in pins and gold sputter‐coated for their visualization.

On the other hand, LSM was used to visualize metabolically active biofilm on the electrode surface. After operation as bioanode, the electrodes were carefully removed from the reactor and fluorescently stained with the LIVE/DEAD BacLight bacterial viability kit (Invitrogen). For this task, 2 μL of a mixture 1:1 of SYTO9: propidium iodide was added to 1 mL of phosphate buffer (90 mM). The electrodes were exposed to this mixture for 15 min at room temperature in the dark before washing with buffer phosphate twice to remove the excess staining. Fluorescence images were captured using an inverted microscope (Nikon, ECLIPSE, *Ti‐S*) so that bacteria with intact cell membranes emit green light. The excitation/emission wavelengths for SYTO 9 and propidium iodide were 488/500–550 nm and 543/600–670 nm. Metabolically active biofilm was observed under different light intensities.

## RESULTS AND DISCUSSION

Expanded graphite PV15 was first activated under different conditions and their physicochemical properties and electrochemical behaviour were characterized before and after activation. Then, all materials were tested in the presence of the model electroactive microorganism electrochemically analysed, and biological assays were carried out to study the influence of the materials' nanopores on their microbial electroactivity.

### Thermal behaviour and CO_2_‐activation of expanded graphite PV15

**FIGURE 2:**
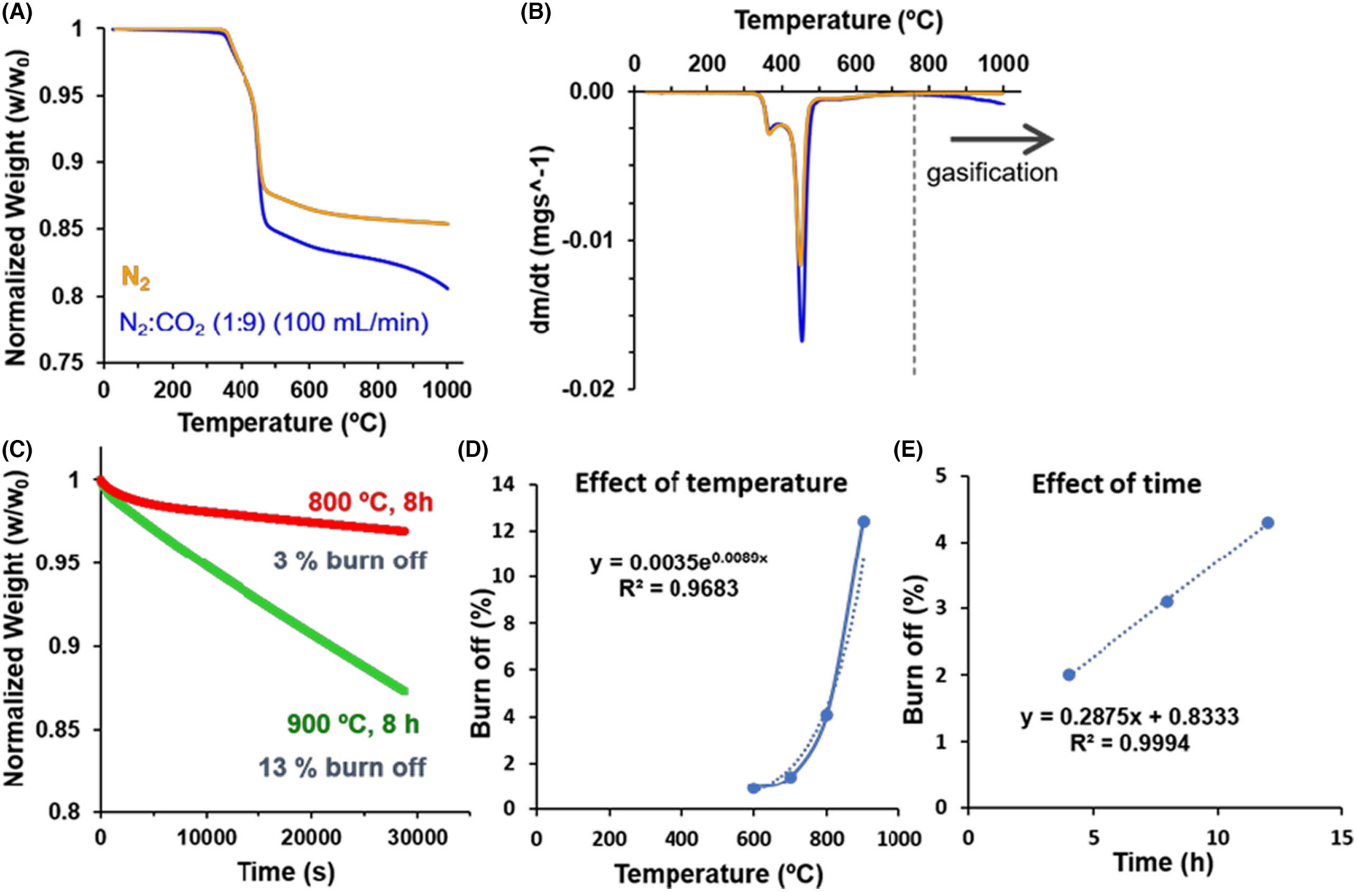
(A) Normalized weight loss and (B) the corresponding derivative (DTG) curves of PV15 in N_2_ and N_2_:CO_2_ (1:9) atmospheres. Gas flow = 100 mL min^−1^. Heating rate = 10°C min^−1^. Effect of temperature at constant time (8 h) on (C) the weight loss and (D) burn off and (E) the effect of time at 800°C on the burn‐off.

The evolution of the PV15 sample weight (normalized) during heat treatment under two different atmospheres, that is, inert (N_2_) or reactive (N_2_:CO_2_), as well as the corresponding derivative curves, are shown in Figure 2A,B, respectively. The matching thermograms (Figure 2A) indicate that the thermal behaviour of PV15 is practically independent of the atmosphere until ca. 450°C. Thus, in both cases, this material decomposes from ca. 330°C up to 490°C, encompassing two overlapped processes (Figure 2B). The first process between 300 and 395°C reaches a maximum decomposition rate at 366°C, whereas the strongest one shows its maximum rate at ca. 450°C. However, both figures evidence that the extent of the decomposition between 450 and 490°C is more marked for the CO_2_ gas. Thus, the weight loss up to this temperature is ca. 12.4% and 14.9% for the N_2_ and CO_2_ atmosphere, respectively. This reflects the higher reactivity of CO_2_ compared with N_2_, even at this moderate temperature range. The weight loss of the material heated in the CO_2_ atmosphere practically coincides with the 15 wt.% of binder polymer in PV15, as provided by the company, so the thermal process between 330 and 490°C is certainly attributed to the decomposition of this polymer on the surface of this material.

Next, from 490 to 700°C, the material experiences a softer decomposition process, with a 1.6% and 1.9% weight loss for the N_2_ and CO_2_ atmospheres, respectively. This process could be related to the release of less accessible inner parts of the polymer alone or pulling out some fragments of graphitic layers on PV15 during decomposition. This is in line with the eruption‐like big holes (1–10 μm) observed by SEM on some parts of PV15 surface (see Figure S2A,B).

Finally, above 700°C, while the weight of PV15 stabilizes in N_2_ gas, it continues decreasing in CO_2_. This weight loss is then ascribed to the gasification of the graphitic material with CO_2_ (see Equation 1) (Contescu et al., 2018), a phenomenon that may start around this temperature at the used conditions.

(1) $$C\left(s\right)+CO_{2}\;\left(g\right)\to 2\;\mathrm{CO}\;\left(g\right)$$

Hence, the effects of the temperature and reaction time on the gasification of PV15 were studied. In these experiments, the weight of PV15 was monitored upon heating up to a given temperature and, subsequently, during different isothermal conditions (see some examples in Figure 2C). Table 2 collects the burn‐off values (%) of the different samples calculated as the weight‐loss percentage during these isothermal conditions. In addition, the table also includes the isothermal oxidation rates of PV15 (expressed as variation of weight loss per time, Δwt(%)/Δt(min)) deduced from the obtained burn‐off values divided by the corresponding studied reaction times.

**TABLE 2:** Burn‐off (BO) and oxidation rate (OR) calculated at isothermal conditions and textural properties (from gas adsorption) of PV15 and some derivatives obtained under different conditions.

Electrode^a^	B.O.	O.R.	A_BET_	*V*_0.995_	*V*_DR_ (N_2_)	*V*_meso_	A_DR_ (CO_2_)	*V*_DR_ (CO_2_)
	%	% min^−1^	m^2^ g^−1^	cm^3^ g^−1^	cm^3^ g^−1^	cm^3^ g^−1^	m^2^ g^−1^	cm^3^ g^−1^
PV15	—	—	0.0	0.000	0.000	0.000	9	0.003
PV15 (N_2_)	—	—	94	0.101	0.038	0.063	157	0.067
PV15‐600‐8h	0.8	0.002	83	0.112	0.031	0.081	115	0.049
PV15‐700‐8h	1.7	0.004	82	0.147	0.033	0.114	88	0.038
PV15‐800‐8h	5.1	0.011	88	0.152	0.036	0.116	106	0.046
PV15‐900‐8h	12.4	0.026	40	0.209	0.016	0.191	44	0.019
PV15‐800‐4h	3.1	0.013	83	0.149	0.033	0.116	83	0.036
PV15‐800‐12h	7.1	0.010	93	0.178	0.037	0.141	111	0.048

^a^ The obtained samples are referred to as *PV15‐T–t*, where *T* is the temperature (in °C), and *t* is the holding time (in h) in the isothermal treatment.

In general, the relatively low BO and OR values found for PV15 (Table 2) are ascribed to the slowness of gasification reaction and therefore, high stability, of graphitic structures in PV15. Nevertheless, both the BO as well as the OR of PV15 increase with temperature and time. Particularly, an exponential increase in burn‐off and oxidation rate with temperature is observed (Figure 2D). This behaviour can be generally represented by the Arrhenius relationship (see Equation 2), in agreement with that observed for other carbonaceous materials during CO_2_ oxidation (Contescu et al., 2018).

(2) $$\text{rate}\;\left(\frac{\%}{\min}\right)=\frac{\mathrm{\Delta wt}\left(\%\right)}{\Delta t\left(\min\right)}=A\exp\left(-\frac{E_{\mathrm{act}}}{\mathrm{RT}}\right)$$

where Δwt/Δ*t* is the rate of weight loss by chemical reaction at constant temperature *T* (K), *R* is the gas constant (8.314 J mol^−1^ K^−1^), *E*_act_ is the activation energy and *A* is the pre‐exponential factor. From the linear representation ln Δwt/Δ*t* versus 1/*T*, the calculated apparent kinetic parameters for PV15 were *E*_act_ = 79 kJ mol^−1^ and *A* = 71.6 min^−1^. These values agree with those of other carbon materials found in the literature (Contescu et al., 2018).

On the other hand, at constant temperature, the burn‐off of PV15 increases linearly with time (Figure 2E). This behaviour has been observed by other authors (Bergna et al., 2019; Rodríguez‐Reinoso et al., 1995; Rodríguez‐Reinoso & Molina‐Sabio, 1992) and clearly reflects that the gasification rate for the studied material and conditions is constant.

### Characterization of expanded graphite PV15 and CO_2_‐activated derived samples

**FIGURE 3:**
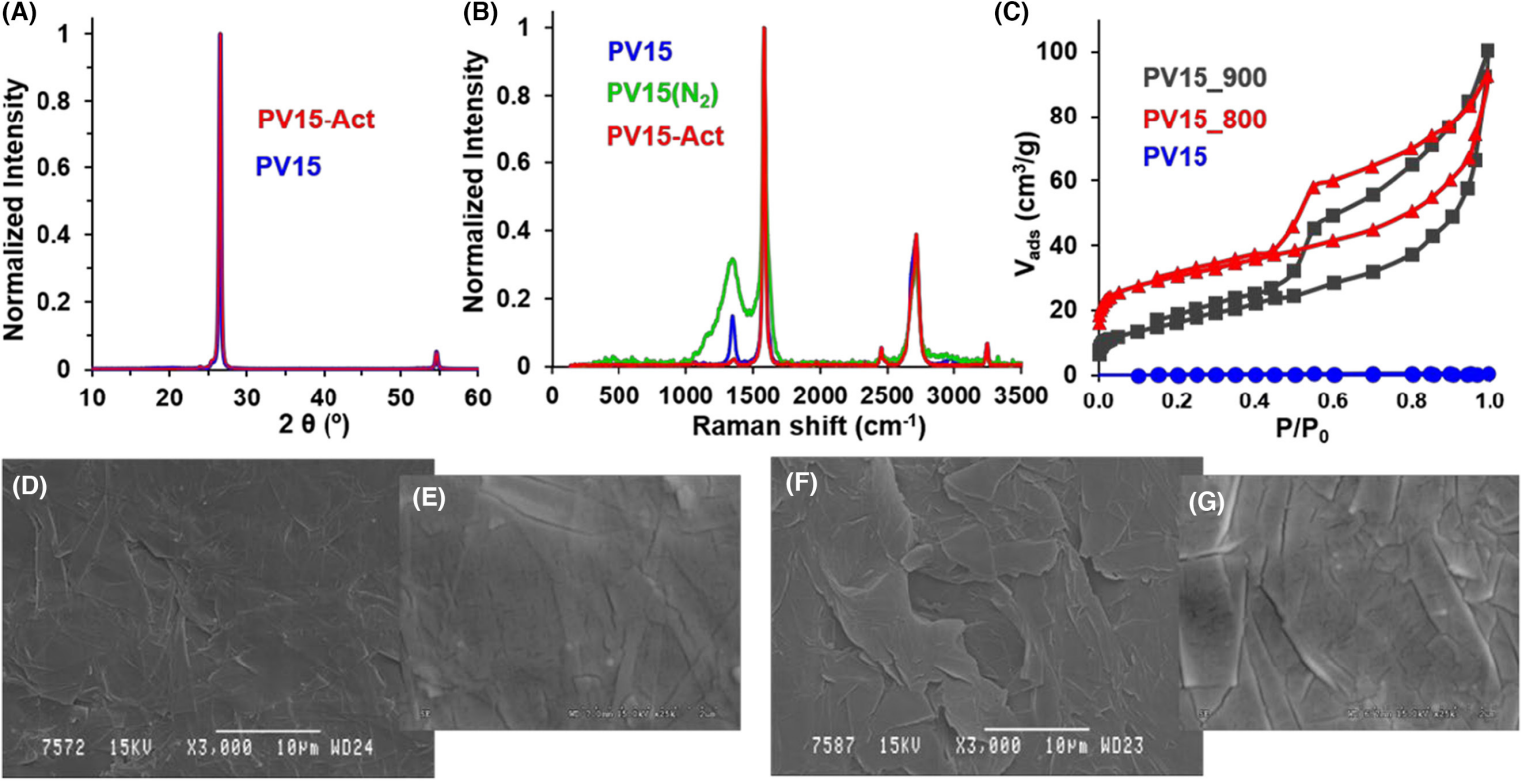
(A) XRD and (B) Raman spectra, (C) N_2_ adsorption–desorption isotherms at −196°C of different samples, and (D–G) SEM images of PV15 (D, E) and PV15‐800‐8h (F, G).

From the structural point of view, the observed high intensity and remarkably narrow XRD peaks of PV15 (Figure 3A) stress the high crystallinity of this carbonaceous material. These peaks, centred at around 2θ = 26.55 and 54.65°, are related to the vertical or horizontal arrangement of graphene sheets aligned along the (002) or (100) planes in graphite, respectively (Coutinho et al., 2000; Rodríguez‐Mirasol et al., 1996). Further details on the crystallinity of this material are provided in the SI (Table S2). Because of such a graphitic structure, PV15 exhibits a high conductivity of 123 S cm^−1^ (0.008 Ω cm), which makes it suitable as a current collector in electrochemical devices.

In parallel, two strong bands centred at 1584 and ~ 2719 cm^−1^ in the Raman spectrum of PV15 (Figure 3B), the so‐called G and 2D bands, have been assigned to the degree of two‐ and three‐dimensionally graphitic orientation, respectively (Rodríguez‐Mirasol et al., 1996). Particularly, the Raman shift, high relative intensity and narrowness of these bands found for this material (Table S2) are also characteristic of a high degree of structural order (Cuesta et al., 1994; Wang et al., 1990). Nevertheless, the so‐called D band at 1348 cm^−1^ is indicative of surface structural defects on this graphitic material. In this sense, chemical analysis by XPS evidenced F atoms (15.3 at.%) and aliphatic carbon bonds on PV15 surface (Table S3), confirming the presence of the polyfluorinated binder polymer on this material.

**FIGURE 4:**
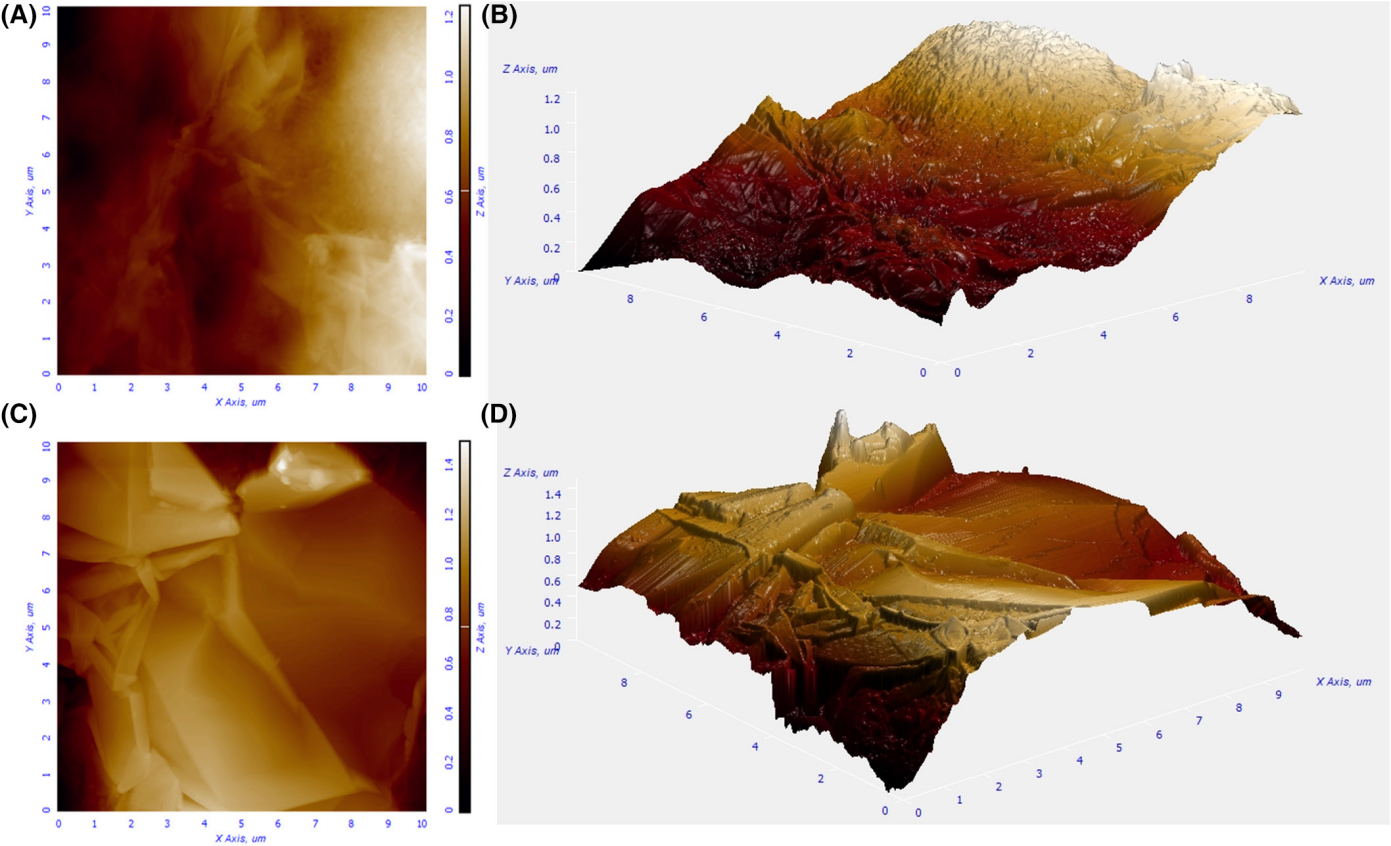
2D and 3D AFM images of PV15 (10 × 10 μm) before (A, B) and after (C, D) CO_2_ activation.

Respect to the textural features, the null N_2_ and CO_2_ adsorptions on pristine PV15 (Figure 3C and Table 2) stress the smoothness of this material at the narrowest nano‐scale. Moreover, SEM images evidence the overall flat surface of this material at microscale (Figure 3D,E). However, the images also show some cracks of 20–50 nm width and laminates on this material. Hence, AFM was used to get further insight into the topography of this sample. Despite apparently flat, the 2D (Figure 4A) and 3D (Figure 4B) AFM images reveal unevenness of up to 1.2 μm and certainly some roughness on the PV15 surface. Specifically, the calculated roughness average (Ra) and root mean square roughness (Rq) were 85.952 and 103.934 nm, respectively.

Interestingly, the practically identical X‐ray diffractograms (Figure 3A) and quite similar G and 2D Raman bands (Figure 3B) observed for PV15 before and after heating in different atmospheres indicated that its inner graphitic structure and therefore, electrical conductivity [140 S cm^−1^ (resistivity = 0.007 Ω cm) for PV15‐800‐8h], was not significantly affected by the studied thermal treatments. By contrast, the thermal treatments were found to affect mainly the surface of this material. Thus, XPS pointed out that F atoms were released during thermal treatment in both N_2_ and CO_2_ atmospheres (Table S3), and Raman and textural characterization highlighted significant differences between the samples heated in these two distinct gases.

On the one hand, while the D band practically vanishes in the case of the CO_2_‐activated sample, the relative intensity and width of this band remarkably increased for the sample heated under N_2_ atmosphere (Figure 3B). Since this contribution is ascribed to the fluoropolymer film, the obtained results suggest that the surface may be cleaned and practically free of defects when heated in CO_2_, but a pyrolysed decomposition product seems to remain after treatment in N_2_ gas.

On the other hand, the different heat treatments were found to greatly develop the textural properties of PV15 (Table 2). The observed IV‐type shape of N_2_ adsorption–desorption isotherms (Figure 3C) reveal the formation of micropores and mesopores (Rouquerol et al., 1994) during heat treatment. These small pores are generally assigned to the spaces left empty among graphitic foils by the release of the binding polymer and/or the oxidative reaction of graphite with CO_2_ (Equation 1).

Nonetheless, other phenomena could also contribute to the formation of these small pores. Thus, despite showing the lowest weight loss (i.e. the lowest degree of polymer removal), the sample treated under N_2_ gas up to 1000°C (PV15(N_2_)) was found to develop the largest volume of ultramicropores (0.067 cm^3^ g^−1^) and among the largest volumes of micropores (0.038 cm^3^ g^−1^) in the present study (Table 2). Since this sample still contains a residue of the carbonized polymer (as deduced from TG and Raman), this higher microporosity may be ascribed to the formation of pores and/or cracks in the polymer film itself by the partial decomposition and/or release of polymer molecules. In fact, the presence of porous and rough deposits on PV15(N_2_) was confirmed by SEM and AFM (see Figures S2B,C and S3). In addition, the incomplete polymer decomposition could also explain the second‐largest ultramicroporosity found for the CO_2_‐derived sample obtained at the lowest temperature studied (PV15‐600‐8h in Table 2). However, the initial stages of CO_2_ gasification should not be ruled out.

As deduced from Table 2, the increment in temperature from 600 to 900°C and/or time generally increased the total volume of pores (*V*_0.995_), that is, the degree of activation, on PV15. However, the pore structure was greatly affected by the chosen heating conditions. On the one hand, the volume of ultramicropores first decreased to reach a minimum for the sample obtained at 700°C; but it subsequently increased for the sample 800°C and it drastically decayed when obtained at 900°C. On the other hand, while the volume of mesopores steadily increased with temperature, the volume of micropores increased up to 800°C and it remarkably dropped for the sample prepared at 900°C (from 40% to 20% of the total pore volume). On the other hand, at a constant temperature of 800°C, the increase in reaction time progressively augmented the volume of ultramicro‐, micro‐ and mesopores, at least for the first 12 h of isothermal treatment.

The minimum ultramicroporosity found for PV15‐700‐8h suggests the absence or minimisation of porous deposits on this sample and, therefore, the promoted or complete decomposition of the binder polymer from 700°C. Next, a higher temperature like 800°C may concurrently favour the generation of ultramicropores, and their subsequent widening into micropores and/or mesopores on the graphitic layers by CO_2_ gasification (see Equation 1). Afterwards, further pore widening seems to be promoted at 900°C, increasing the relative proportion of mesopores. These results point out the interconversion of ultramicropores into micropores and that of micropores into mesopores with increasing temperature and reaction time (Rodríguez‐Mirasol et al., 1993).

Concerning the surface morphology and roughness of CO_2_‐activated samples (see PV15‐800‐8h as an example), both SEM (Figure 3F,G) and AFM (Figure 4C,D) images clearly show that heat treatments in CO_2_ efficiently remove the binder polymer to expose the interconnected graphite sheets on the surface of PV15. This is in line with Raman and XPS analyses. Moreover, unlike the case of the treatment in N_2_ gas, big holes are not observed in the CO_2_‐derived samples, so the reaction with CO_2_ might facilitate the release of less accessible polymer chains. From the analysis of AFM images, the calculated Ra and Rq for PV15‐800‐8h were 153.208 and 204.800 nm, respectively. These average‐like parameters are approximately twice those found for pristine PV15 and reflect the more abrupt topography resulting from the exposition of graphite sheets (Figure 4D).

The obtained results demonstrate that both CO_2_ gas and temperatures high enough are necessary to eliminate the polymer from the PV15 surface. Although the surface became more abrupt, the average roughness (ca. 85–150 nm) did not seem so remarkable, at least with respect to the micron‐sized electroactive bacteria. By contrast, the main effect of the CO_2_ reaction has been found the activation of the PV15 surface, that is, the generation of a large volume of pores ranging from 0.7 to 50 nm. These pores are too small, that is, physically inaccessible for an electroactive bacteria like *Geobacter*, whose dimensions comprise ca. 1–3 μm. Then, the influence of these changes on the microbial electrochemical response was studied. Because of the cleanliness and larger volume of small pores, the samples prepared at 800 and 900°C were selected for this study.

### Microbial electroactivity

#### Abiotic control

The first step for studying microbial electroactivity on the surfaces of the different materials was to perform CV on each of the working electrodes before inoculating the reactor. This task was carried out with three purposes: (1) to corroborate the optimal connection of the electrodes with the current collectors (wires); (2) to ensure that there were no electron transfer signals with the electrode surface at the beginning of the experiment when electroactive bacteria were not present and finally, (3) to study the electrochemical change in the surface of commercial material after CO_2_ activation.

**FIGURE 5:**
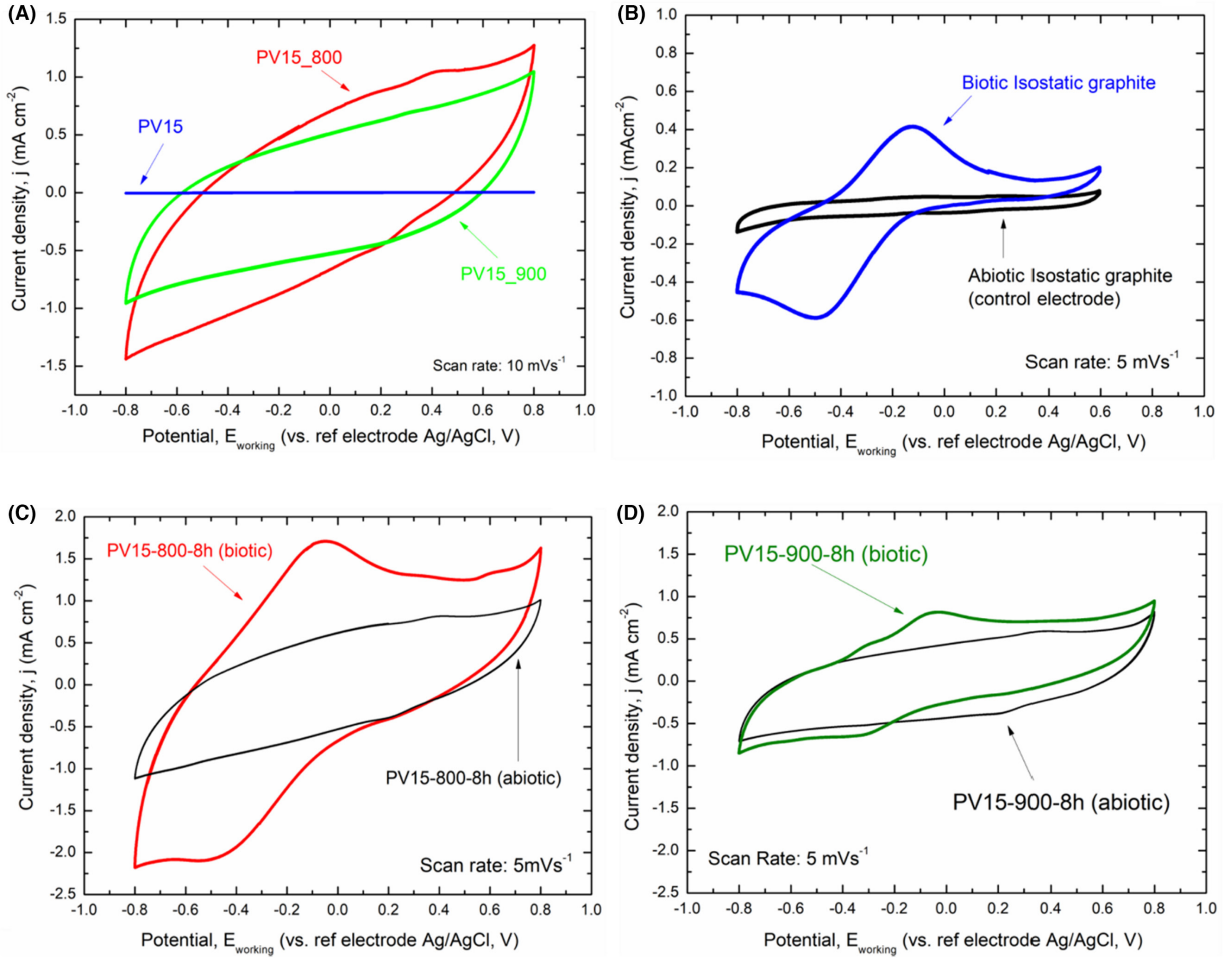
(A) CV for commercial expanded graphite, PV15 (blue line), and activated expanded graphite electrodes at a temperature of 800°C during 8 h (PV15‐800‐8h, red line) and 900°C during 8 h (PV15‐900‐8h, green line) in freshwater media (without *Geobacter sulfurreducens*). CV Scan rate = 10 mV s^−1^. (B) Abiotic initial CV (without *Geobacter sulfurreducens*, black line) and biotic CV after the chronoamperometric experiment (growth of the electroactive biofilm on the surface of the electrode in the presence of *Geobacter sulfurreducens*, blue line) of the control electrode formed by isostatic graphite plate (scan rate = 5 mV s^−1^). (C, D) CV in FWM without *Geobacter* (black line) and after the chronoamperometric experiment with electroactive biofilm on the activated electrodes (C) biotic PV15‐800‐8h (red line) and (D) biotic PV15‐900‐8h (green line) (scan rates = 5 mV s^−1^).

The voltammetric response of the pristine and activated electrode surfaces (Figure 5A) revealed no peak of current intensity in the potential window analysed for PV15; furthermore, no species participated in electron transfer with the surface under these initial conditions. In contrast, the green and red voltammetric cycles corresponding to the activated material PV15‐900‐8h and PV15‐800‐8h, respectively, present a marked capacitance compared to plain PV15 (i.e. without activation), related to double‐layer charging current due to the capacitive‐like nature of the electrode/electrolyte interface, and revealing a higher microporosity structure of the electrodes. The CV curves showed a more rectangular shape in the potential windows, which indicated a better current response behaviour than non‐activated material (since the current depends on the electrode surface area). In order to maximize the capacity of a material, a well‐balanced between meso and microporosity is needed (Fuertes et al., 2005). The charge increases markedly after material CO_2_ activation, and it is associated with the adsorption of ions in micro and mesopores. The adsorption of ions in micropores is more effective than in larger pores due to confined micropores forcing ions to desolvate partially or entirely (Simon & Gogotsi, 2008). Probably, for this reason, the capacity shown by the material activated at 800°C for 8 h was somewhat higher compared to that of the material activated at 900°C for 8 h. In addition, the slightly more tilted CV of PV15‐800‐8h compared to that of PV15‐900‐8h may be attributed to the slightly lower resistance of the former material, which shows a lower activation (burn‐off) degree.

The electrochemical behaviour in the freshwater medium of the abiotic non‐activated expanded graphite papers (PV15 and Papyex), isostatic graphite plate, and activated PV15 electrodes through the CV technique is shown in SI (Figure S4) for comparison purposes. PV15 voltammetric profile is very similar to Papyex (Figure S4A) and even to that of the control isostatic graphite plate, although it had a more significant double layer (Figure S4B). The more significant double layer of the activated expanded graphites compared to the isostatic graphite plate was also confirmed (Figure S4C).

#### Biological assays: Microbial current generation

**FIGURE 6:**
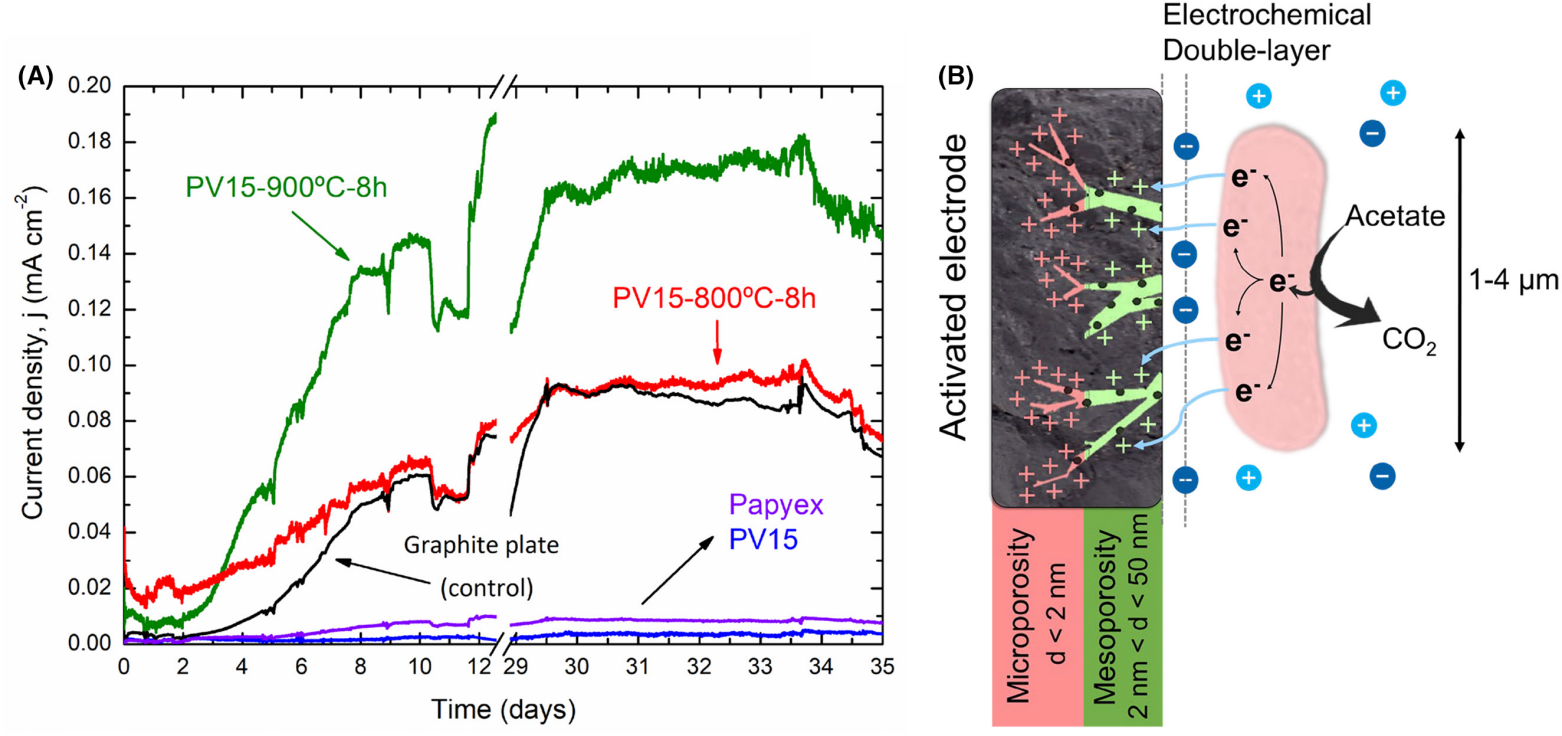
(A) Current density (mA cm^−2^) generated by *Geobacter sulfurreducens*. Working electrodes were operated at a constant potential of 0.2 V versus Ag/AgCl, 3 M NaCl reference electrode. (B) Proposed effect of nano‐scale porosity in the microbial extracellular electron transfer. The size difference between a bacteria (1–4 μm) and nano‐scale pores (nm) are represented.

The objective of this section was to analyse the response of the activated and non‐activated materials to the growth of electroactive biofilms of *Geobacter sulfurreducens*. In this sense, we classified the quality of the material according to the current density generated by the electroactive biofilm. The electrons were generated during the oxidation of an organic substrate (i.e. acetate) by electroactive bacteria. The bioreactor was designed to house all the working electrodes in the same physicochemical and biological conditions. All working electrodes were polarized at 0.2 V (vs. Ag /AgCl, 3 M NaCl reference electrode), and the current was recorded for 35 days in the presence of *Geobacter sulfurreducens* inoculum (Figure 6A) (see Supplementary Information for abiotic controls and experiment repetitions to ensure reproducibility, Figures S5A,B and S6A,B). It is noteworthy to highlight that this bioelectrochemical reactor is not an MFC. Instead, it is a practical system to study and compare the performance of electroactive bacteria on different materials.

Chronoamperometry showed two explicit scenarios. Firstly, in non‐activated expanded graphite materials, an increase in current density was not observed due to the growth of the *Geobacter sulfurreducens* film. However, an increased current was observed during experiments with activated materials, PV15‐800‐8h and PV15‐900‐8h, and the control material. As explained before, these currents are related to the different microbiological activity towards acetate oxidation in the aqueous solution (medium). The chronoamperometry of these last materials (activated and electrode control) showed two clear phases of the growth of the electroactive biofilm. The lag phase (the first 2 days) provided a current density almost negligible. The freshwater medium (FWM) circulation through the reactor (*t* = 48 h) renewed the medium and a second phase was observed. Thus, the current density was increased exponentially, indicating a constant growth of the electroactive biofilm on the surface of the materials. After several days, a stable current density was reached, being *j* = 0.09 mA cm^−2^ for the activated electrode PV15‐800‐8h, and *j* = 0.17 mA cm^−2^ for the activated electrode PV15‐900‐8h (red and green line in Figure 6A, respectively). This current density value indicated steady‐state biofilm formation (the growth and death and/or electroactivity of the bacteria were constant). According to the literature, the steady‐state current density depends on many parameters such as electrode material, temperature and biofilm composition, and it is considered a feature of a particular electroactive electrode/biofilm system (Logan, 2008). Table 3 summarizes the steady‐state current densities for the studied electrodes and compares them with other reported studies under similar experimental conditions.

**TABLE 3:** Summary of current densities for the studied electrodes and other reported studies using a three‐electrode set‐up and polarized at 0.2 V versus Ag/AgCl.

Anode material	Pore size	Inoculum	Substrate	*j* (mA cm^−2^)	Refs.
Carbon fibre	6.8 μm	Wastewater	Acetate 10 mM	*j* (max) = 3.0	He et al. (2011)
	0.4 μm			*j* (max) = 1.7	
Commercial carbon felt	47 μm	Wastewater	Acetate 10 mM	*j* (max) = 1.2	Chen et al. (2011)
NCP‐CFM	38 μm			*j* (max) = 0.5	
2D‐ECFM	0.6 μm			*j* (max) = 0.17	
Carbon felt	20–200 μm	Sludge	Food waste	*j* (max) = 0.3	Blanchet et al. (2016)
Non‐porous ITO	‐	*S. oneidensis*	–	*j* (max) = 0.00005	Wenzel et al. (2018)
Polystyrene microspheres	80–140 μm			*j* (max) = 0.03	
Nanoparticle suspension (nanoporous)	10–100 nm			*j* (max) = 0.006	
Papyex (Mersen)	*–*	*G. sulfurreducens*	Acetate 20 mM	*j* (steady‐state) = 0.008	This study
PV15 (SGL Carbon)				*j* (steady‐state) = 0.003	
PV15‐800‐8h	<50 nm			*j* (steady‐state) = 0.10	
PV15‐900‐8h				*j* (steady‐state) = 0.17	

Abbreviations: 2D‐ECFM, Electrospun‐carbon nanofiber; ITO, non‐porous indium tin oxide; NCP‐CFM, Natural cellulose paper – Carbon nanofiber mat.

Interestingly, introducing micro‐ and mesoporosity in PV15 improves biofilm activity (60‐fold) compared to plain EG. The electrode activated at 800°C (for 8 h), with the largest volume of micropores, reaches almost the same steady‐state current as the control electrode. However, the electrode activated at 900°C with many micropores and a higher volume of mesopores reached ca. 0.2 mA cm^−2^ (in steady state), which is almost double the current density provided by previous electrode materials. Even if the microbial current density could be slightly lower compared to the electric current provided by reported materials in the literature (see Table 3), the use of these activated materials could significantly decrease the internal resistances (i.e. high costs associated with the use of the state‐of‐the‐art electrodes in METs.

A similar improvement in bioelectricity production has been observed in systems where the anode material in the presence of carbon nanotubes (CNTs) improved the extracellular electron transfer (Ma & Hou, 2019), as microporosity was increased after modification. In this work, the authors claimed that the carbon nanotube‐chitosan (CNT‐CS) layer with mesoporous and microporous structure provides a strong interaction with microbial films (Xie et al., 2011), facilitating electron transfer between biofilm and the conductive surface. However, the nature of this strong interaction was not described or defined.

Despite this work is focused on the study and demonstration of the phenomenon itself (neither it has been intended nor the experiments have been designed to study its mechanism), potential reasonable explanations can be suggested. Thus, taking into account that micro and mesoporosity in the material are not accessible to microorganisms, a possible hypothesis for this improvement in current production could be that the number of electrons exchanged by electroactive microorganisms could be determined by the number of surface charges that must be compensated by ion adsorption on the electrode pores (Figure 6B). In this sense, the highest current density produced by bacteria has been observed for the electrode prepared at the highest activation temperature (PV15‐900‐8h), which shows the largest number of total pores (*V*_0.995_ in Table 2). Nonetheless, this bioelectrode also exhibits the highest proportion of mesopores (*V*_meso_ in Table 2), so not only micropores but also bigger nanopores might play an important role. In fact, it is well known that mesopores are essential for faster ion diffusion into electrode inner micropores to produce larger currents in distinct electrochemical applications (Liu & Creager, 2010; Momodu et al., 2017; Zhang et al., 2014b). On the other hand, previous studies in abiotic media showed that the adsorption of proteins, like *cit c*, is promoted in nanostructured carbon films with increasing pore sizes between 30 and 150 nm (Vijayaraj et al., 2010). Hence, given the fact that *cit c* is considered to be a protein involved in direct EET by electroactive bacteria (Busalmen et al., 2008), it is proposed that the presence of mesopores or bigger nanopores in the studied activated samples may somehow facilitate the physicochemical interaction and/or EET with *Geobacter*'s proteins.

The electrochemical characterization of biofilm development on the electrode surfaces during experiments was carried out by CV. Figure 5 compares the biofilm evolution on the surface of the activated electrode PV15‐800‐8h (Figure 5C) and PV15‐900‐8h (Figure 5D) before inoculation of the bioreactor (black line) and at the steady‐state (constant current density in the chronoamperometry) (coloured line). The same procedure was carried out with the control electrode (Figure 5B). The CV at steady‐state current density showed the characteristic turnover signal obtained when the extracellular electron transfer capacity (EET) of electroactive bacteria occurs (in the presence of an electron donor) (Maestro et al., 2014). The signal showed a redox couple attributed to the electron transfer from the electron donor (acetate) to the carbonaceous surface, mediated by the C‐type cytochromes of the electroactive bacteria (Prado et al., 2019; Richter et al., 2009).

The difference between the CVs of colonized materials was remarkable. The activated electrode PV15‐900‐8h revealed the highest steady‐state current density during chronoamperometry, but a lower response in current density value throughout the entire potential window analysed. However, this current density value was higher for the activated material PV15‐800‐8h, which showed a lower current density during the biofilm growth experiment. This effect could be due to a higher surface area of the PV15‐800‐8h material (Table 2), which contains a larger volume of micropores on its surface (i.e. effect of the electrical double‐layer capacity of the modified electrode due to surface area increase).

**FIGURE 7:**
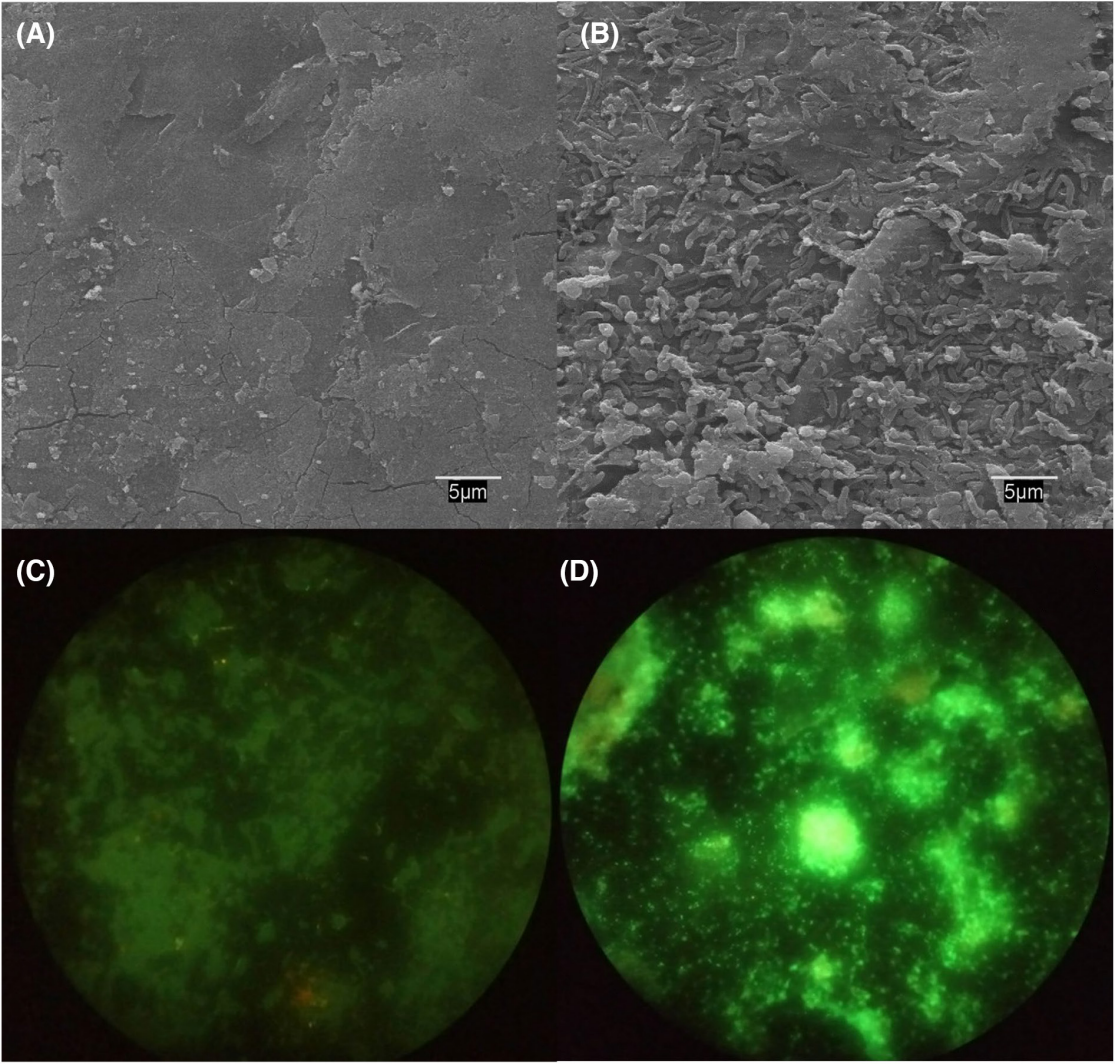
Verification of biofilm growth: SEM images of the surface of the activated electrode PV15‐800‐8h, (A) before and (B) after the chronoamperometry in the presence of electroactive bacteria; and fluorescence (LSM) images of the same electrode (C) before and (D) after this chronoamperometric experiment. Bacteria with intact cell membranes stain green.

After the electrochemical analysis, the materials were duly prepared for their visualization with SEM and LSM microscopic techniques. The obtained SEM and LSM images of the activated electrode PV15‐800‐8h before the biotic chronoamperometry showed the absence of any biofilm on its surface (Figure 7A,C, respectively). On the contrary, after the chronoamperometric experiment, the same electrode surface exhibited a clear biofilm distinguishing the characteristic bacillus shape of *Geobacter sulfurreducens* (Figure 7B). Moreover, the presence of green fluorescence clusters of live bacterial cells was observed in the LSM image of this electrode after biological assays (Figure 7D). Similar images were obtained for the activated electrode PV15‐900‐8h. These results corroborate the formation of a *Geobacter sulfurreducens* biofilm on the electroactive electrode surfaces.

## CONCLUSIONS

This work presents a systematic study on the CO_2_ activation of PV15 commercial expanded graphite including microbial electroactivity responses in the presence of this upgraded material. Thus, this contribution tackles two poorly studied but interesting topics in MET, that is, the effect of nano‐scale porosity in the response of electroactive bacteria and the potential use of EG as bioelectrode. The obtained results indicate that PV15 gasifies in the CO_2_ atmosphere from 700°C, progressively increasing the volume and mean diameter of nanopores with the temperature and reaction time. Apart from the changes in textural properties and the removal of binder polymer, these treatments do not significantly affect the microstructure and electrical conductivity of PV15.

Voltammetric characterization of the materials under abiotic conditions reveals that CO_2_ activation causes a huge increase in the electrical double layer capacitance (EDLC) of PV15 (up to 425 times) as the main electrochemical consequence of nanopores generation. In addition, under biotic conditions, this technique also evidences that the extracellular electron transfer (EET) of *Geobacter sulfurreducens* on PV15 was greatly promoted after CO_2_ activation. Furthermore, chronoamperometries and microscopy analysis have demonstrated that CO_2_ activation treatments greatly promote the growth and bioelectricity production (up to 60 times) of *Geobacter sulfurreducens*.

From (i) the consistency of nanopores generation and remarkable EDLC enhancement, (ii) the insignificant modification of other properties as well as (iii) the inaccessibility of bacteria to the created nanopores; the observed effective redox coupling between *Geobacter* and CO_2_‐activated PV15 samples points out a direct effect of nanoporosity on microbial electroactivity. It is proposed that the capability of electroactive microorganisms to transfer electrons with carbon surfaces may be greatly affected by the availability of sites (nanopores) to accommodate or compensate electric charge in the electrode surface. Moreover, these pores could also promote the interaction or EET with bacteria proteins ranging nanoscale dimensions. Nevertheless, the understanding of the mechanisms of this promoted activity needs further studies.

The present research not only presents a new strategy to enhance the performance of bioelectrodes but it also suggests that activated EG electrodes could be good candidates for the simplification and cost reduction of different bioelectrochemical systems.

## AUTHOR CONTRIBUTIONS

**M. Ramírez Moreno:** Conceptualization (equal); investigation (equal); methodology (equal); writing – original draft (equal). **R. Berenguer:** Conceptualization (equal); investigation (equal); supervision (equal); writing – original draft (equal). **J.M. Ortiz:** Investigation (equal); methodology (equal); resources (equal); writing – review and editing (equal). **A. Esteve Núñez:** Resources (equal); validation (equal); writing – review and editing (equal).

## CONFLICT OF INTEREST STATEMENT

There are no conflicts to declare. All authors contributed equally to the work.

## Supporting information


Appendix S1
