# Supplementary material for: Study of the influence of nanoscale porosity on the microbial electroactivity between expanded graphite electrodes and Geobacter sulfurreducens biofilms
Source: Microb Biotechnol. 2023 Dec 27;17(1):e14357. doi: 10.1111/1751-7915.14357 (PMC10832559; doi:10.1111/1751-7915.14357)
Supplement: Supplementary file 1 — Appendix S1 [file MBT2-17-e14357-s001.docx]

Supporting Information

Study of the Influence of Nanoscale Porosity on the microbial electroactivity between expanded graphite electrodes and *Geobacter sulfurreducens* biofilms

M. Ramírez-Moreno^1,2^, R. Berenguer^3, *^_,_ J.M. Ortiz^1^ and A. Esteve-Núñez^1,2,*^

^1^Bioelectrogenesis Group, Instituto Madrileño de Estudios Avanzados IMDEA-Agua, Parque Tecnológico de la Universidad de Alcalá, 28805 Alcalá de Henares, Spain

^2^Universidad de Alcalá, Departamento de Química Analítica, Química Física e Ingeniería Química, 28801 - Alcalá de Henares, Spain

^3^Instituto Universitario de Materiales, Departamento de Química Física, Universidad de Alicante, Apartado 99, 03080-Alicante, Spain

*Corresponding Author

Dr. Raúl Berenguer (email: [raul.berenguer@ua.es](mailto:raul.berenguer@ua.es))

Tel: +34-965909150 / Fax: +34-965903464

Prof. Abraham Esteve-Núñez (email: [abraham.esteve@uah.es](mailto:abraham.esteve@uah.es))

Tel: +34-918854950 / Fax: +34-918855088

Table of Contents:

1. **Assembly and connections of the electrochemical bioreactor.**
2. **Characterisation of PV15 and derived CO_2_-activated samples**
3. **Comparison of microbial electroactivity: commercial materials versus activated materials**
4. **Assembly and connections of the electrochemical bioreactor**


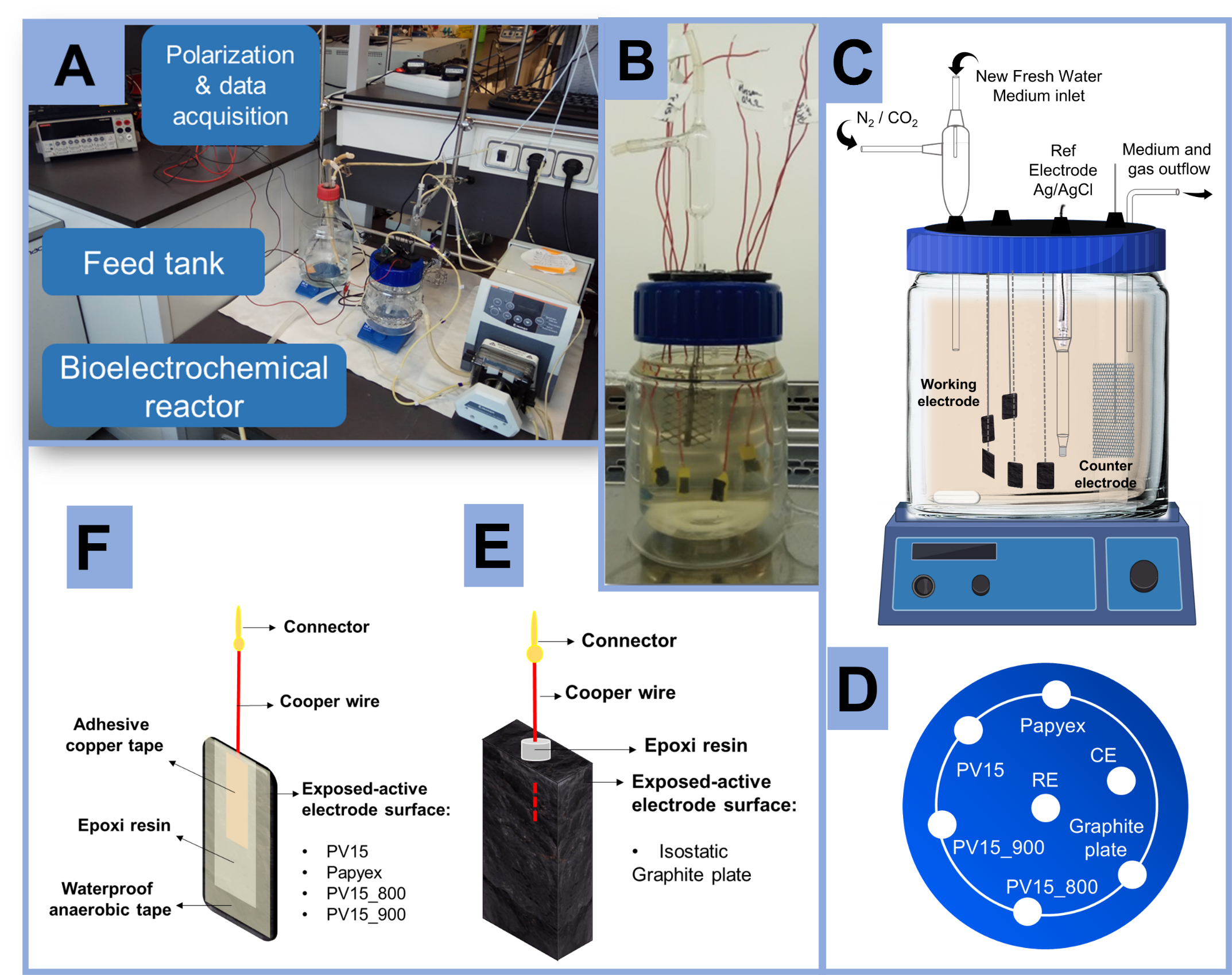


**Figure S1. A)** Photo of the laboratory system setup, from right to left: peristaltic pump, bioreactor, feed tank, Keithley multimeter. **B)** Photo of the 3-electrode bioreactor in the sterilization chamber. **C)** Diagram of the 3-electrode bioreactor and the assembly of the different electrodes. **D)** Scheme of the bioreactor top cap and the position of the different working electrodes made with different materials. CE = counter electrode, RE = reference electrode. **E)** Type of connection mode for the control electrode based on isostatic graphite plate. **F)** Type of connection made for working electrodes based on expanded graphites (activated and non-activated).

**Table S1.** Materials used as working electrodes in this work, showing details of their geometric areas and electrode-connection resistances.

| Working Anode Electrode | Type Material | Enterprise | Geometric Area Electrode  (cm^2^) | Resistance with connection (Ω) |
| --- | --- | --- | --- | --- |
| PV15-800ºC-8h | Activated EG | - | 0.6 | 2.4 |
| PV15-900ºC-8h | Activated EG | - | 0.6 | 3.7 |
| PV15 | Commercial EG | SGL-Carbon | 3.6 | 1.8 |
| Papyex | Commercial EG | Mersen | 3.6 | 1.7 |
| Control electrode | Isostatic Graphite plate | Mersen | 11.54 | 1.3 |

1. **Characterisation of PV15 and derived CO_2_-activated samples**


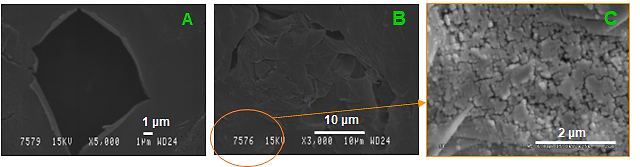


**Figure S2**. SEM images showing big holes (1-10 μm) (**A,B**) and porous deposits (**B,C**) on PV15(N_2_) surface.

**Table S2**. Crystallinity (position of the 002 and 100 peaks, and calculated crystallite dimensions of the graphite in the vertical (La) or horizontal (Lc) directions) and microstructure (Raman shift and width of the G and D bands) from XRD and Raman characterization, respectively.

|  | **XRD** | | | | **Raman** | | |
| --- | --- | --- | --- | --- | --- | --- | --- |
| **Electrode** | ***002***  **(2θ)** | ***Lc***  **(Å)** | ***100***  **(2θ)** | ***La***  **(Å)** | ***ν*_G_**  **(cm^-1^)** | ***Δν*_G_**  **(cm^-1^)** | ***Δν*_D_**  **(cm^-1^)** |
| **PV15** | 26.540 | 388 | 54.666 | 389 | 1583.6 | 21.5 | 41.5 |
| **PV15(N_2_)** | nd* | nd | nd | nd | 1584.5 | 141.8 | 35.4 |
| **PV15-800-8h** | 26.540 | 343 | 54.667 | 373 | 1583.9 | 23.4 | 36.6 |

*nd = non determined

**Table S3**. Surface chemical composition (wt. %) of some PV15 samples determined by X-ray photoelectron spectroscopy (XPS).

| **Electrode** | **C** | **O** | **F** |
| --- | --- | --- | --- |
| **PV15** | 80.1 | 4.6 | 15.3 |
| **PV15(N_2_)** | 98.1 | 1.9 | --- |
| **PV15-800-8h** | 99.1 | 1.0 | --- |


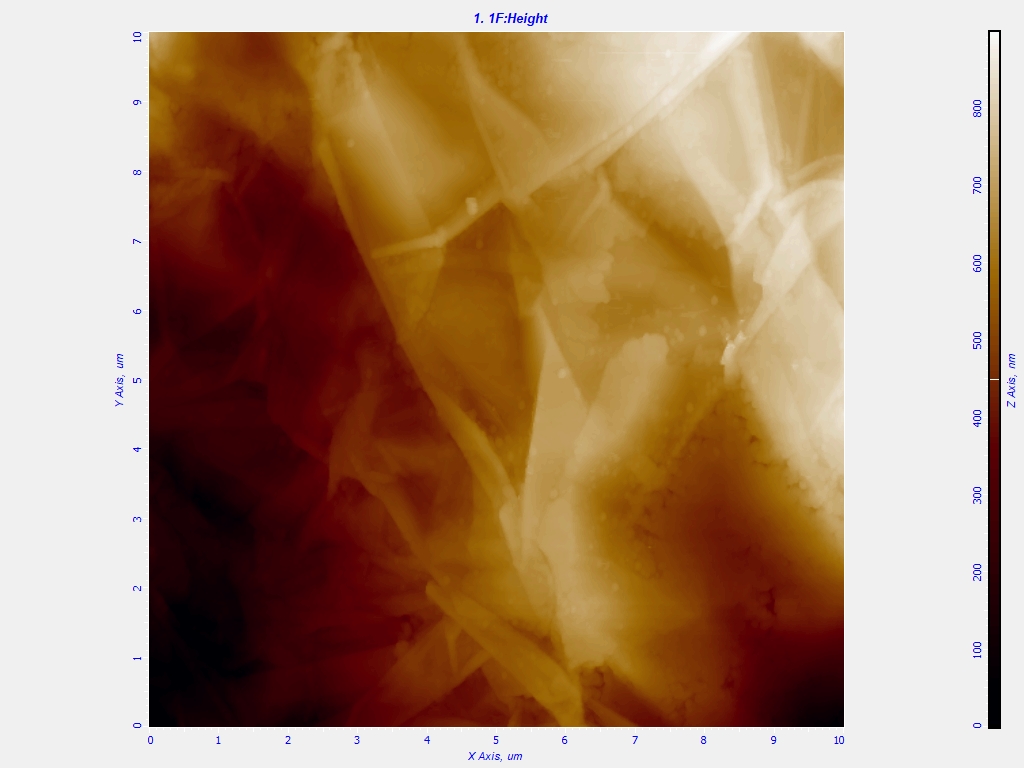


**Figure S3**. 2D AFM image of PV15(N_2_) which shows the presence of undecomposed deposits.


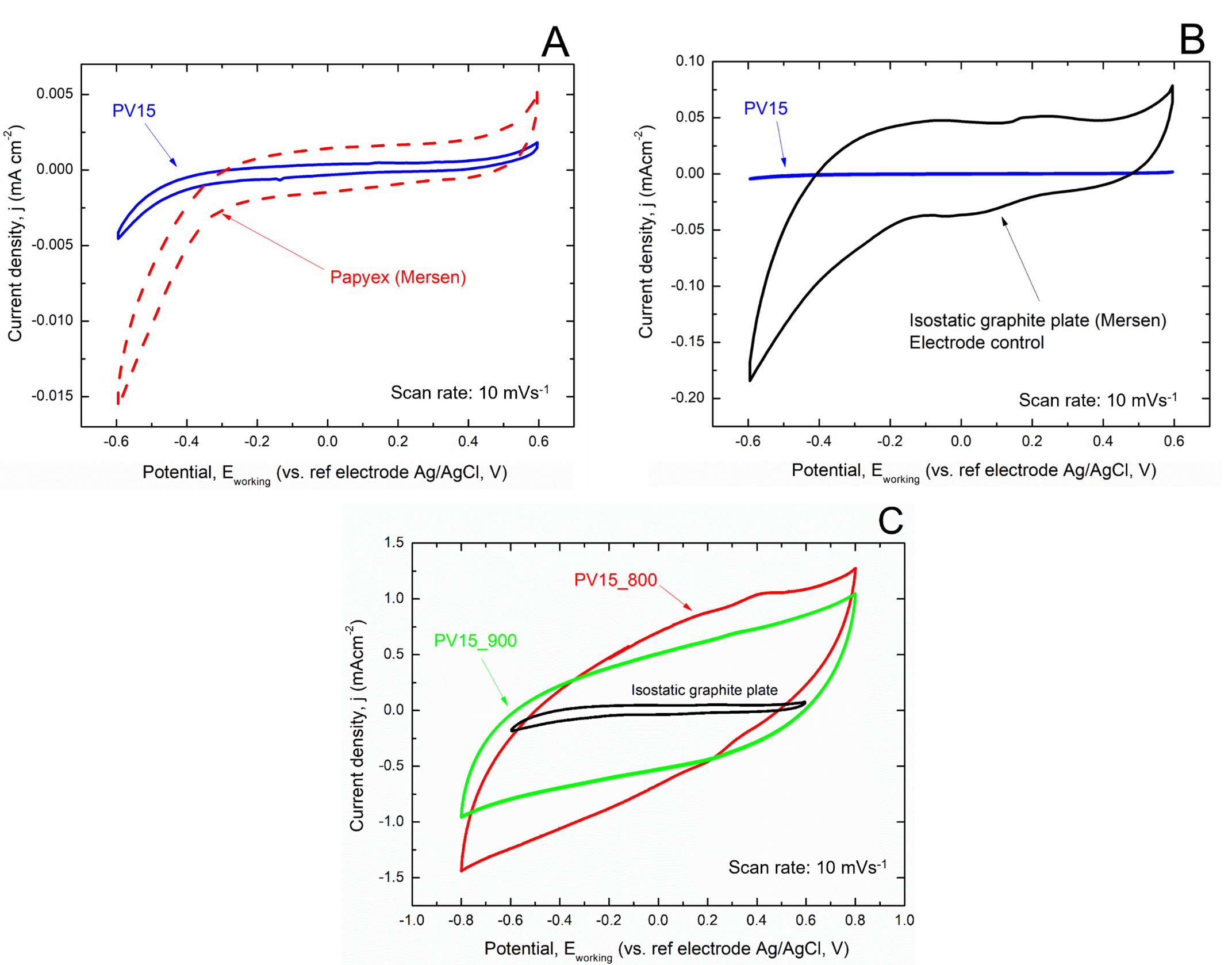


**Figure S4.** Abiotic initial cyclic voltammetry (without bioreactor inoculation) performed in FWM for: **(A)** PV15 electrode (blue line); Papyex electrode (red dash). **(B)** PV15 electrode (blue line) and control electrode, isostatic graphite plate (black line). **(C)** Isostatic graphite plate as control electrode (black line) versus activated expanded graphite paper PV15-800-8h (red line) and PV15-900-8h (green line).

1. **Comparison of microbial electroactivity: commercial materials versus activated materials**







**Figure S5.** Chronoamperometry at 0.2 V (vs. Ag /AgCl, 3M NaCl reference electrode) to perform the abiotic tests of the activated materials **(A)** PV15-800-8h **(B)** PV15-900-8h in the freshwater medium during 6 days approximately. **(A)** Current density (biology response) from electrobacteria after inoculation of the cell for PV15-800-8h (red line) and abiotic signal for the same system without electroactive bacteria (black line). **(B)** Current density (biology response) from electrobacteria after inoculation of the cell for PV15-900-8h (green line) and abiotic signal for the same system without electroactive bacteria (black line).







**Figure S6.** Chronoamperometry during the repeated experiment to study microbial electroactivity on the surfaces of activated electrodes. **(A)** Chronoamperometry with the polarization (0.2 V vs. Ag /AgCl, 3M NaCl reference electrode) of the electrode composed of the activated material PV15-800-8h. **(B)** Chronoamperometry with the polarization (0.2 V vs. Ag /AgCl, 3M NaCl reference electrode) of the electrode composed of the activated material PV15-900-8h.
